# Artificial Teeth

**Published:** 1870-12

**Authors:** W. M. Herriott

**Affiliations:** Zanesville, O.


### ARTIFICIAL TEETH.

BY W. M. HERRIOTT, ZANESVILLE, O.

Read before the Ohio State Dental Society.

As our Executive Committee made no provision for discussing the subject of artificial dentures during the present meeting of the Society, I will offer a few thoughts which may at least provoke others to think.

In estimating the relative merits of substitutes for natural dentures, attention is first directed to their greater or less resemblance to the organs they are intended to replace. To arrive at an intelligent decision, comparison should be instituted with the varied and characteristic developments of the teeth in both sexes, in different temperaments, and at various periods of life, for unless the physiological laws which determine the relations of size, shape, color, etc., have been recognized by the manufacturer, his stock will not offer opportunity for the selection of teeth to meet the different needs of patients.

Of cource *vital* appearance is an absolute necessity and must be maintained as much in *artificial* light as in day-light, and depends upon the color, texture, translucency, etc. The colors of the body and enamel, though differing, should be nicely—almost imperceptibly—blended; the texture soft and waxy, and a translucency varying to suit varying ages and temperaments.

The next point I desire to fix attention upon is *form*, individual and relative. Each tooth should have "character," and in the set the relation of one to each and each to all should be such as to give the best artistic effect. Nature's differences are distinctive and characteristic, looking not merely to use but indicating physical, mental and mora peculiarities.

In the respects named the artificial teeth offered to us by S. S. White will stand comparison with the natural teeth, and contrast favorably with those of any other manufacturers, and they are also superior, because of the ease with which, from their conformation, they can be adapted to the various conformations of the maxillary, and made to antagonize, so as to produce the best results for speech and mastication, and as in any test of strength, the weight must be taken into account, they are found to have *strength* without bulk or weight. As no piece of mechanism is stronger than its weakest part, it is the object to preserve such a relation between the tooth substance and the pins as to shape, size and angle of insertion, that one will be as strong as the other and both sufficient for all legitimate uses.

It will be seen by examining the teeth of S. S. White that in the "foot shaped" pin the wire is "upset" so that the thickest part of the pin is at the heel or angle, the point or toe running upward into the thick part of the tooth, giving additional security against its being drawn out. A decided gain has also been made by him in the direction or angle in which the pin is inserted in the tooth, avoiding weakening the thin portion of the tooth, as is done when the headed pin is inserted in a straight line, and giving the greatest amount of resistance where the greatest strain is brought to bear upon the surface in biting and masticating.

His teeth also bear a close resemblance to the natural organs in the general and most noticeable features, such as shade, texture, translucency, individual and relative shape and position. Superiority over all other makers of teeth has in short been gained by him in all that goes to make up that which is desired by the dental operator in supplying artificial for natural teeth.

I shall next consider bases upon which to mount these teeth. Without stopping to say anything concerning porcelain, continuous gum or gold, all of which have done good service when properly used by skilled operators in constructing artificial dentures, I will say that we have greater need of some lower priced material.

To meet this want a number of bases have been presented of different metals, and different compounds of the same metals. But as a single metal is, if pure, to be preferred to an alloy, aluminum plates cast would appear to be the desideratum, it having great power to resist the oxidizing action of air, etc., equal to platinum. It also resists the action of nitric and sulphuric acids when cold; it does not, however, resist chlorine. It is hard but malleable; has ductility and great tenacity. Same bulk being one-fourth the weight of silver, it is well suited when weight is an objection, and its conductibility makes it peculiarly well adapted for dental plates. But we have not had any process presented to us for casting perfect plates of aluminum, unless it be that offered to the profession by Dr. Feemster, of Greencastle, Indiana, who is perfecting a process which we hope will overcome the obstacles heretofore existing. Next among the alloys of which tin makes the largest part, we find Watt & Williams' metallic base the best; for when compared with others in the market it has a better color, runs sharper, finishes easier, is stronger, is less liable to shrink, and resists chemical action better. Concentrated nitric acid will dissolve it; so will hydrochloric acid, when aided by heat; and the teeth being well supported by it a very *thin* plate will give sufficient strength.

It is very pure, containing no lead, antimony, cadmium, zinc, nickle, copper, mercury, or any other poisonous metal. To conclude, an artificial denture formed by properly mounting S. S. White's teeth upon Watt & Williams' metallic base gives us the best lower priced artificial denture ever used to supply the place of the natural denture.

